# The Benefit of 3D Laser Scanning Technology in the Generation and Calibration of FEM Models for Health Assessment of Concrete Structures

**DOI:** 10.3390/s141121889

**Published:** 2014-11-19

**Authors:** Hao Yang, Xiangyang Xu, Ingo Neumann

**Affiliations:** Geodetic Institute, Faculty of Civil Engineering and Geodetic Science, Leibniz University Hanover, Nienburger Street 1. D-30167, Hanover, Germany

**Keywords:** TLS, FEM, point cloud, surface based, concrete, calibration

## Abstract

Terrestrial laser scanning technology (TLS) is a new technique for quickly getting three-dimensional information. In this paper we research the health assessment of concrete structures with a Finite Element Method (FEM) model based on TLS. The goal focuses on the benefits of 3D TLS in the generation and calibration of FEM models, in order to build a convenient, efficient and intelligent model which can be widely used for the detection and assessment of bridges, buildings, subways and other objects. After comparing the finite element simulation with surface-based measurement data from TLS, the FEM model is determined to be acceptable with an error of less than 5%. The benefit of TLS lies mainly in the possibility of a surface-based validation of results predicted by the FEM model.

## 1. Introduction

### 1.1. Terrestrial Laser Scanning

**Figure 1.**
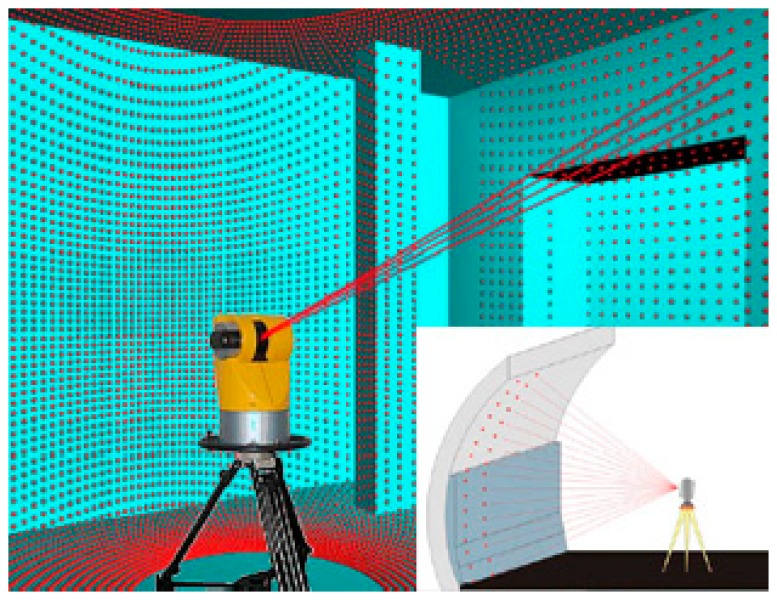
Sampling process with TLS (Adapted from [3]).

Terrestrial 3D laser scanning technology (TLS) is a relatively new technique for quickly getting three-dimensional spatial information. It was hailed as another technological revolution in the field of surveying and mapping after GPS technology which accurately reconstructs the scanned objects and builds high-fidelity, high-precision 3D point clouds [1]. TLS helps sample complex objects easily using 3D point clouds. TLS consists of three steps, which are inclination of laser pulses changed by a reflecting mirror, reflection of laser pulses on the surface of objects and reception of reflecting laser signals [2]. Through such processes, TLS is able to acquire a dense 3D coordinate information effectively and precisely over the entire objects or surfaces. TLS technology with high reliability, precision and good flexibility has broad application prospects. At present, the TLS technology is widely used in high-precision ground information, three-dimensional measuring and three-dimensional digital design. The sampling process with TLS is shown in Figure 1.

### 1.2. Significance of the Contribution

The structural health monitoring of structures, *i.e.*, bridges, is an extremely important issue. We should keep in mind that their average age and traffic (especially truck traffic) are continuously increasing. One geodetic task is to provide measurement and evaluation methods of the important physical parameter in close collaboration with other disciplines like civil engineering [4].

Nowadays, construction is developing very fast in the entire world and concrete is one of the most important building materials and is widely used in many types of engineering structures. According to statistics, e.g., China's real estate investment increased from 8 billion Euro in 1991 to 1 trillion Euro in 2013, representing an 125-fold increase in just 20 years. Due to this fast development, the average age of the buildings is just about 20 years. If we consider the time from 1998 and 20 years later, a large amount of the real estate will enter the high-risk period, meaning that a growing number of objects will become scary high-risk buildings after 2018. Nowadays China is in the development stage of urbanization, regardless of whether the market or government will pay more and more attention on security issues. Thus, an intelligent and efficient monitoring and prediction system will have a very great potential for development. Additionally, the use of measurements for more valuable models (like FEM models) is mandatory.

### 1.3. Research State of the Art

Recently, TLS has been often used in various fields such as civil engineering or archeology for object modeling, geographic information system and so on. However, applications for system identification and structural health monitoring are in the beginning stage [5]. Rosser *et al.* [6] used TLS to monitor changes on coastal cliff faces. The results demonstrated that terrestrial laser scanning can be used to quantify cliff failures to a previously unobtainable precision. Monserrat *et al.* [7] monitored land deformation using repeated TLS scans and estimated the deformation parameters using local surface matching. It is interesting to note that the results were achieved under non-optimal conditions, e.g., using non-calibrated data and sub-optimal targets from the matching viewpoint. Park *et al.* [8] presented health monitoring of structures using TLS and adopted a displacement measurement model to improve the accuracy of the measurements.

Surface-based TLS measurements have been reported by many authors. Some authors convert point clouds into a consistent polygonal or mesh, such as in [9]. Vertices, edges and faces are contained in this mesh surface. Tsakiri *et al.* [10] used planes fitted to point clouds when estimating the deformation of a sea-lock. The plane model is appropriate for small regions and therefore the segment was divided into raster cells. In a tunnel monitoring, Van Gosliga *et al.* [11] modelled the tunnel with a cylinder. Chang *et al.* [12] developed a structure surface analysis program. The surface data such as the degree of deformation is acquired easily by statistic regression and polynomial function. Koch [13] fitted a three dimensional NURBS surface [14] by a lofting method. It is shown that the lofting method for estimating the control points and their simultaneous estimation gives identical results for time-dependent problems. The use of TLS in the field of calibration and validation of FEM is new; e.g., [15]. For this reason, this issue will be addressed in this paper. Many techniques and devices for acquiring 3D information have been developed in recent years [16–19]. As terrestrial laser scanners have become more available, their applications have become more widespread, creating a demand for affordable, efficient and user-friendly devices [20–23]. Several studies have analysed the behaviour of these instruments [24–27].

FEM is a good choice for analyzing problems over complicated domains, when the desired precision varies over the entire domain, or when the solution lacks smoothness. FEM as applied in engineering is a computational tool for performing engineering analysis. It includes the use of mesh generation techniques for dividing a complex problem into small elements, as well as the use of software programs coded with FEM algorithms [28]. FEM is a numerical technique for finding approximate solutions to boundary value problems for differential equations. It uses variational methods (the calculus of variations) to minimize an error function and produce a stable solution. “Analogous to the idea that connecting many tiny straight lines can approximate a larger circle, FEM encompasses all the methods for connecting many simple element equations over many small subdomains, named finite elements, to approximate a more complex equation over a larger domain” [28]. In order to improve the data process, adaptive Kalman-filtering techniques can be used in terms of a realistic model calibration [29].

### 1.4. Framework

This paper compares the FEM model with experimental data on the behavior of concrete beams. Concrete structures are commonly designed to satisfy certain serviceability and safety criteria. On the one hand, experimental research supplies the basic information for finite element models, such as material properties. The development of reliable analytical models can reduce the number of required test specimens for the solution of a given problem, recognizing that tests are time-consuming and costly and often do not simulate exactly the loading and support conditions of the actual structure [30,31].

**Figure 2.**
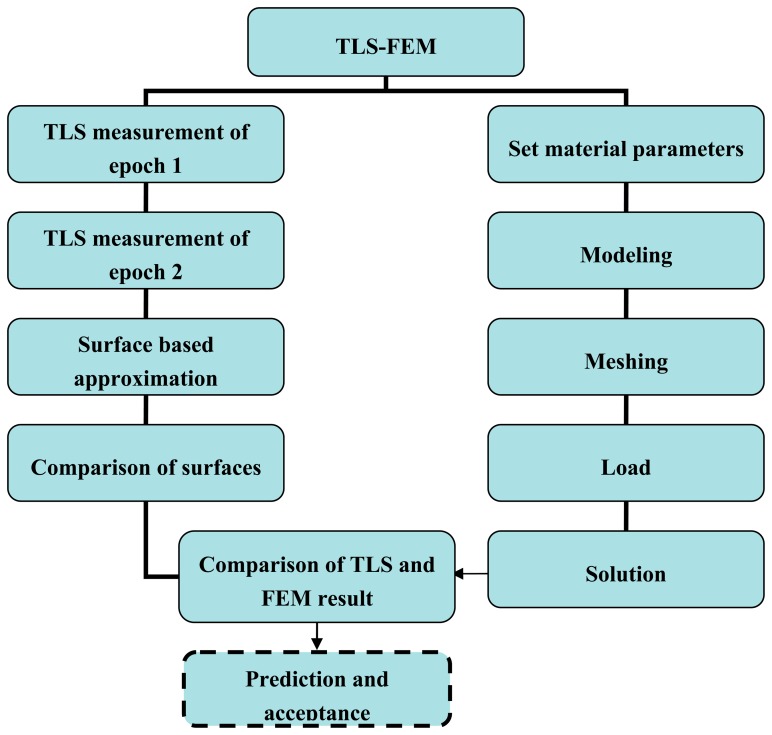
The workflow of TLS and FEM model comparison.

On the other hand, the TLS measurement has lots of benefits and can't be replaced. It offers surface information of object with a high accuracy, rapid measurement and efficiency. The surface-based measurements can be compared with the predictions of a FEM model. In addition, the results of FEM models have to be evaluated by comparing them with experimental data. Within the framework of developing an advanced FEM model analysis method for modern structures, the need for experimental research continues. In Figure 2 we present the workflow in details.

In Figure 2 the left side is the TLS experiment which contains the measurement of epoch data, surface-based approximation and surface difference analysis (see Section 2); the right side is the FEM model analysis which includes parameters setting, modeling, meshing, load and solution (see Section 3). With the comparison of the TLS measurement and FEM model simulation, we will obtain an acceptable FEM model. In the future, the prediction and acceptance of FEM models will be present in the next step, so we draw a dotted line here.

## 2. Experimental Setup and Data Analysis

**Figure 3.**
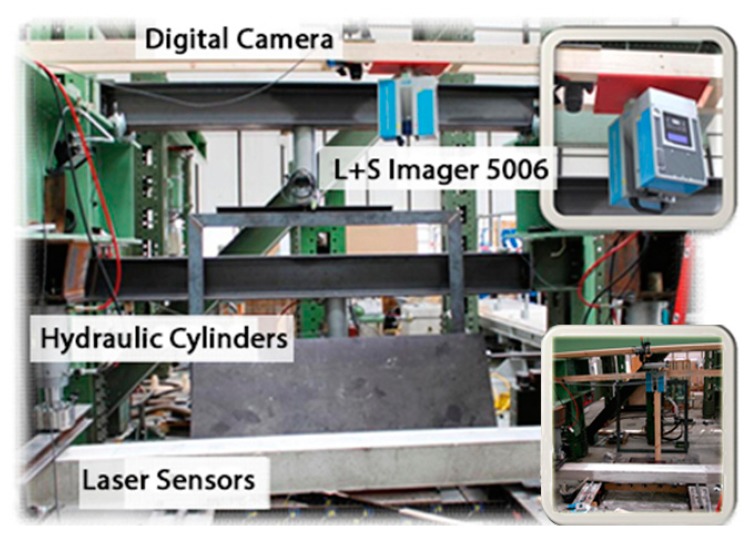
Experiment setup and test (Adapted from [15]).

This experiment (see Figure 3) was applied in order to observe cracking, displacement and other intricate concrete structural effects. The loading method involved two cylinders fixed at both ends of a specimen [15].

**Figure 4.**
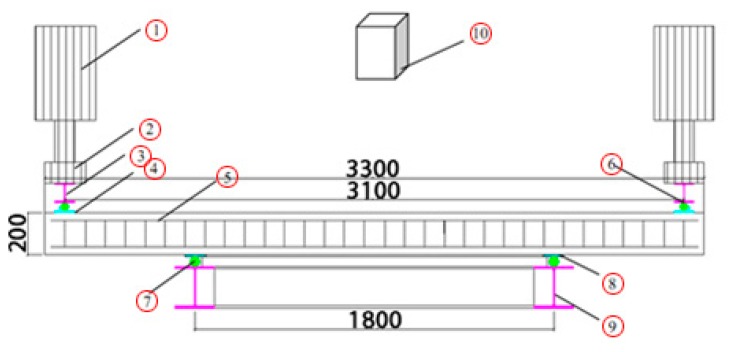
Introduction of the test setup (Adapted from [15]). The Nos. in the picture are listed here: 1. Hydraulic jack; 2. Force sensors; 3. HEB 200 I-steel; 4. Steel plate of 5 mm; 5. Test specimen; 6. Steel tube (diameter: 5 mm); 7. Steel tube (diameter: 5 mm); 8. Steel plate of 5 mm; 9. I-steel frame (HEB 200); 10. Laser scanner.

The load increment was selected at 4 kN up to the formation of the first crack and then the load increment was increased to 5 kN. Each load step was held for 5 min. The loading was continued until the ultimate load. The test setup is introduced in Figure 4.

The tested slab-strips were carefully inspected at each load step. The load and deformation were measured by force sensors and laser displacement sensors. The triangular displacement sensors are in the middle position under the beam. The TLS obtains the point clouds of the beam at every load step. We gather the load and displacement data in Table 1. The experimental setup presented here should provide an example for the general working steps and ideas in the paper. For further information about the test see [15].

In the Table 1, the load (force) is added step by step as the second line and the displacement in third line responds to the middle of the beam.

## 3. Surface Based Measurement Analysis of TLS

The TLS result is point-based. We can get the X, Y, Z coordinates and the intensity of a reflected laser beam at each point. However, the points contain noise and aren't therefore individually so accurate. Surface-based measurement analysis overcomes this shortcoming. It is smooth and accurate and reveals the geometric relationshipd between each part of the object. In addition, surface-based methods give information about arbitrary points of the object when they give the X, Y, Z coordinates, while point clouds only show information of scanned points, but no information about the connection between two scanned points. Thus, surface-based methods deserve our attention.

### 3.1. 3D Approximation by Free Form Surface

**Figure 5.**
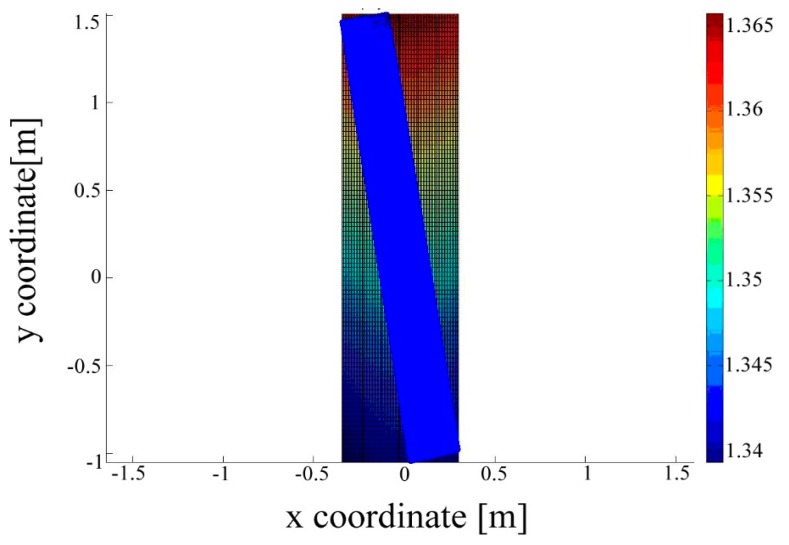
Polynomial approximation of the point cloud.

A free-form surface can be fitted to the heights of an object measured by a laser scanner. The fit can be done by polynomials, B-splines and NURBS [14]. In this paper, we attempt to apply the third-order-polynomial method to the surface of a concrete beam. Due to the fact the scanned data contains large point clouds and the comparison of differences between large datasets is time-consuming, we do an estimation of the point clouds. A recursion algorithm is adopted to improve the efficiency, see e.g., [13]. In every step, 1000 points are fitted and generate the same 10 parameters of the polynomial. Finally, the polynomial surface is drawn in Figure 5.

The point cloud (blue: beam) is tilted, due to the TLS was not fixed against the beam, but with a small rotation (see Figure 3). The color bar corresponds to the height of the polynomial approximation of the point cloud. X axis is the width direction and Y axis is the length direction.

### 3.2. Computing the Deformations

If we approximate the original surface and the surface after deformation, we can easily calculate the changes at arbitrary point and the surface changes. The deformation between two epochs during a load experiment can be calculated with a self-developed program which predominantly takes advantage of polynomial approximation under MATLAB.

**Figure 6.**
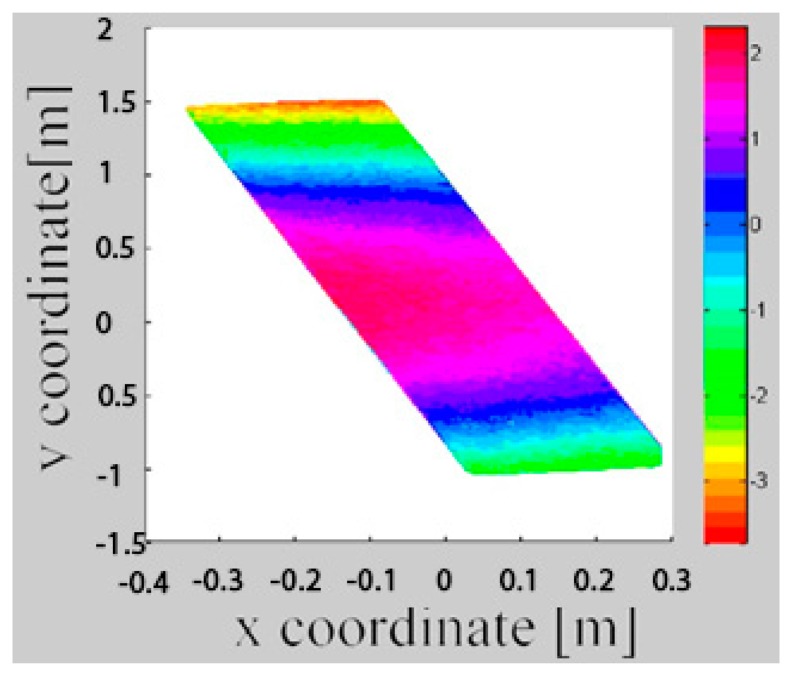
The difference of point clouds between epoch 1 and 2 (Adapted from [15]).

The difference between two epochs is here calculated from the Z-coordinates of the epochs. Therefore, for a given X/Y-coordinate pair, the Z-coordinate in each epoche is computed and then substracted from each other. In this case it is similar to the smallest distance between the surface in the two epochs. The difference of point clouds between the epochs is shown in Figure 6. The unit of X and Y axis is m, but the unit of the color bar is mm. From Figure 6 we can see the noise of the point clouds is obvious. This is why a surface-based approach is superior.

**Figure 7.**
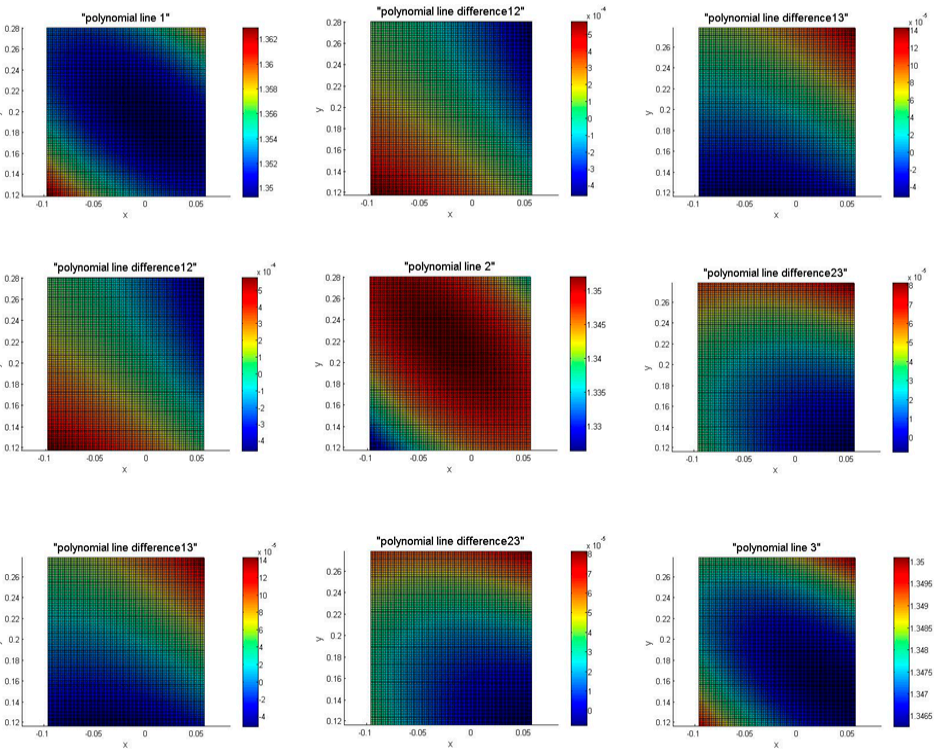
The plots of epoch 1, 2, 3 and their differences.

In order to present the differences in the surfaces, we extract part of the epoch data in Figure 7. The differences show the movements/deformations of the beam under load (see Section 2). In the diagonal are the plots of epochs 1, 2 and 3 (see Table 1), which depict an area of 10 cm × 10 cm from the beam. The plots at the middle of first line are the difference between epoch 1 and epoch 2; the right of the first line is the difference between epoch 1 and epoch 3; the right of the second line is the difference between epoch 2 and epoch 3. The diagonal line, corresponding to polynomials 1, 2 and 3 shows the plots are symmetrical. The difference between the polynomial 1 and polynomial 3 is similar to the difference between polynomial 2 and polynomial 3, and the tops of both plots are positive (red color) and the bottoms are negative (blue color) which reveals that the deformation is not strictly symmetrical (see Figure 7).

In Figure 7, the surface of epochs 1, 2 and 3, fitted by the polynomial approximation, are symmetric in the diagonal direction, but a gradient appears in the anti-diagonal direction (particularly evident in epoch 3). The reason is that TLS is not aligned to the beam but tilted. The surface differences are symmetrical only in the direction of diagonal which means that the beam is twisted when the loads are increasing. Also, because of the small area selection on the beam, the color bars are fairly sensitive to the deformation information. This can lead to the asymmetry, like the difference between epoch 2 and 3. Compared with the point cloud difference (see Figure 6), the max. displacement in Figure 6 is about 2 mm and it is about 0.5 mm in Figure 7. This means that the noise is significantly reduced by the fitting process which smoothes the deformation as expected. The deformation is different between top and bottom surface, due to the fact the displacement measured by TLS, which is fixed at the top of the beam, is smaller than the one measured by the triangular displacement sensor, which is at the bottom of the beam.

### 3.3. Deformation Analysis

After the computation of the deformations, the significance of the results should be judged. Only deformations with a magnitude significantly larger than the measurement uncertainty should be accepted under realistic conditions. For that reason, the surface-based deformations need to be studied for significance. This needs the consideration of the observed uncertainty of the two contributing measured epochs. By applying surface-based techniques in the approximation of the TLS point cloud, the uncertainties can be reduced significantly [15]. Additionally, the determination of deformations is more or less the computation of measurement differences. Therefore, under the same measurement configuration in the contributing epochs, many systematic measurement uncertainties could be reduced or even eliminated. There are some experiences about the results of TLS in deformation of concrete beams [15] where the systematic errors are eliminated when the distance between two surfaces is calculated, e.g., [32]. Any blunders in the measurement can be detected by hypothesis testing. The critical value is the distance between the sampled points and the surface. We compare the distance with the standard deviation from all point distances to the surface. The point is deleted if the distance is larger than approx. 3 times the standard deviation of all distances.

From this point of view, the surfaced-based analysis of the measurements from the TLS is mandatory and one needs to have an optimal measurement configuration. A validation of the accuracy of similar measurements (as the here presented measurements) was done in [15]. The laser triangulation sensors and additional levelling measurements (with an accuracy of about 1/10 of a mm) were carried out and showed an agreement with the surface based smoothed TLS-measurements in a range of a few tenths of a mm. Within the measurement configuration, the user needs to take into account that bad incidence angles of the laser beam on the object surface and non-optimal object surfaces should be avoided in TLS measurements. For further details on the uncertainty of TLS measurements see, e.g., [33].

## 4. FEM Model Establishing

A FEM model of a target structure to be measured is established by meshing the interpolated points. Displacements of all the points can be estimated over the entire surface of the object structure. Using a deformed shape as displacements due to load, strain and stress state of the structure could be evaluted by finite element analysis [34]. Deformation, which is often described as strain, is a change in the shape or size of an object due to an applied force or a change in temperature. Depending on the type of material, size and geometry of the object, and the forces applied, various types of deformation may result.

**Figure 8.**
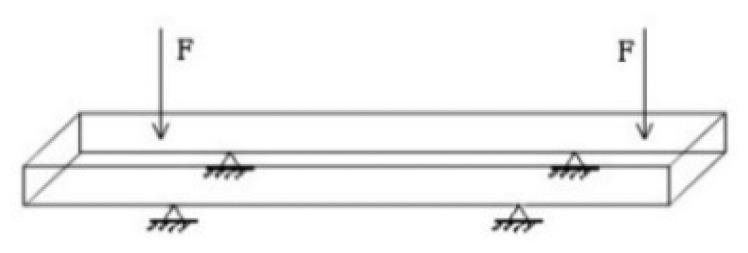
Schematic diagram of concrete bridge FEM model.

In order to simulate the deformation of concrete beam and compare it with the experiment TLS data, we build an exemplary FEM model with ANSYS which can be used for concrete bridges. In the following are some 3D plots with relation to the loads. Since we want to use the epoch data, we set loads as 65 kN. The simplified model of a concrete bridge is shown in Figure 8. The size of the geometric model is 3.3 m × 0.2 m × 0.3 m.

**Figure 9.**
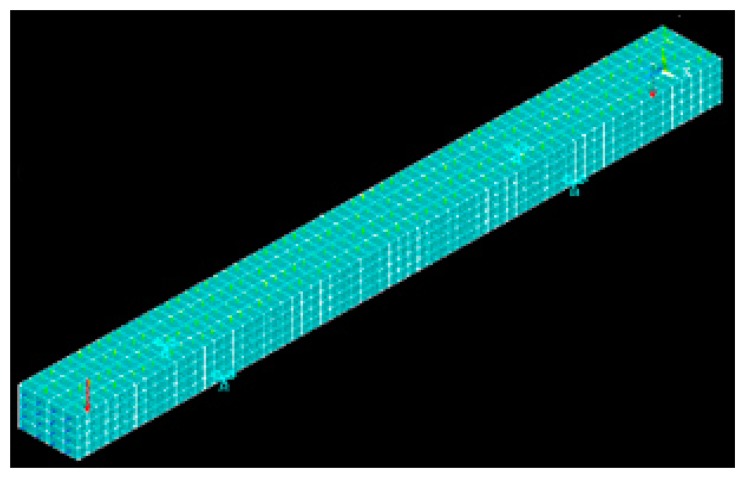
The schematic diagram of FEM model.

We analysed the concrete structure with ANSYS SOLID 65, which includes concrete element data, as the element type. The constraint of displacement is the four supporting points in Figure 8 in the x,z,y directions. The schematic diagram of the FEM model without displacement and force is given in Figure 9.

The parameters are set according to Table 2 where EX stand for elastic modulus; PRXY is Poisson's ratio; ShrCf-Op is Shear transfer coefficient-Open; ShrCf-Cl is Shear transfer coefficient-Close; UnTensSt is uniaxial tensile strength; UnCompSt is uniaxial compressive strength. Material 1 and 2 correspond to concrete and steel, respectively. In this paper, the general idea of the combination of the surface measurements and FEM model should be highlighted. therefore, the detailed FEM model with some other material parameters will be considered in future research. In ANSYS the units of the parameters are MPa, N and mm.

**Figure 10.**
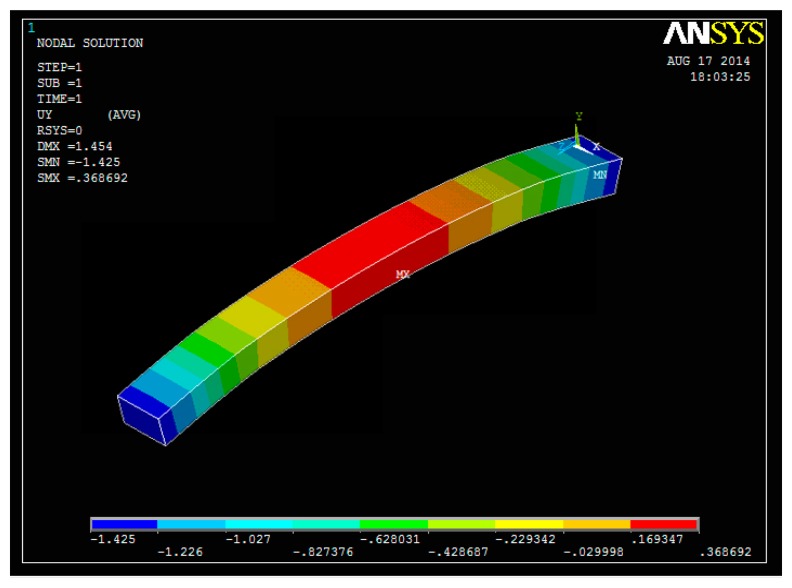
FEM model simulated 3D deformation of epoch 1.

We have two deformation cases: elastic deformation and plastic deformation. The elastic range ends when the material reaches its yield strength, where plastic deformation begins. Following the steps of modeling, meshing, load and solution, we generate a 3D deformation plot of epoch 2 compared with epoch 1, with a force of 14.17 kN. The FEM model simulation of the displacement in the vertical direction is shown in Figure 10.

We focus on the deformation in the middle of the beam, which is shown in red color. Due to the fact the force is symmetric in the FEM model, there is no visible torsion of the beam in the simulation. For better comparison and visual effects, the plot of deformation is amplified in Ansys. The actual deformation is manifested in the values of DMX, SMN and SMX which correspond to the displacement max, solution min and solution max.

## 5. Results and Comparison

In this experiment we analysed nine epoch datasets which have been partly presented in Figure 7. The force and displacement has been listed in Table 1. The epoch 2 is the experiment data with the force of 14.17 kN which will be compared with FEM model (See Figure 12).

**Figure 11.**
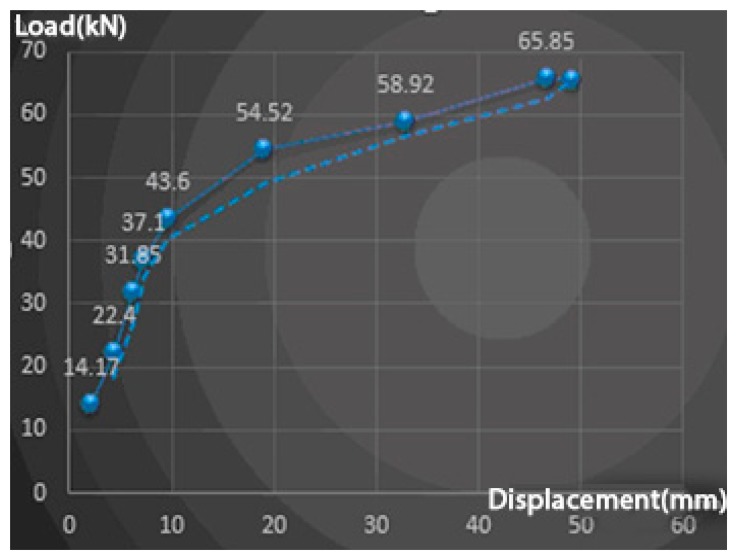
The relation between load and max displacement (middle of beam) of epoch data.

**Figure 12.**
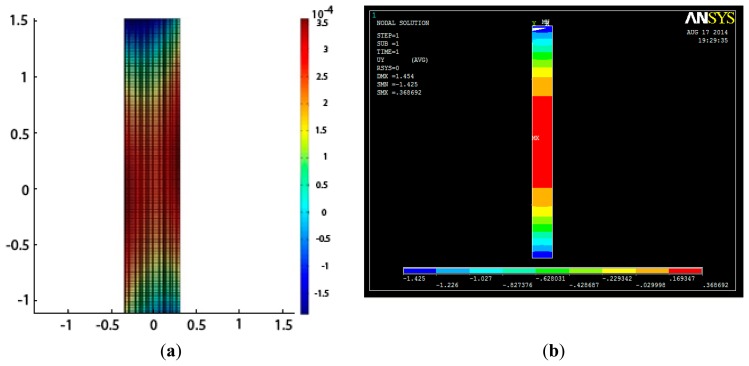
Surface comparison between measurement and FEM model simulation in epoch 2. (**a**). Surface approximation of TLS measurement (**b**). FEM model simulation.

In Figure 11, the y axis is the force with units of kN and the x axis is the displacement with units of mm. We can see an obvious inflection point at the fifth epoch with force 43.6 kN which should be related to the yield strength. After the data has been analysed (see Section 3) and the FEM model is simulated (see Section 4), we compare this FEM model with experimental data epoch 1 as in Figure 12 which not only presents the relationship between displacement and force of single point, but also compares the point distribution on the surface.

**Figure 13.**
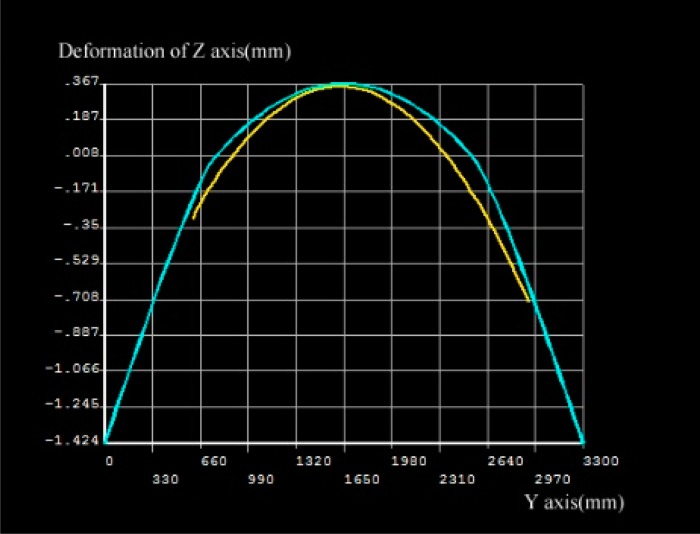
Contrast of vertical displacement between TLS measurement and FEM simulation.

The left plot in Figure 12 is from the TLS measurements and the right one is from the FEM model simulation. The max. displacement of the experimental data between epoch 1 and 2 is around 0.35 mm (see the color bar of the left plot) and that in the FEM model is approximately 0.36 mm. It means the error is less than 5%, which is acceptable. In the left side of Figure 12, two sides of the beams, which is about 200 mm in length, are not in the area of the laser scanner, because of the shield of the hydraulic jack (see Figure 4). In order to compare the deformation between TLS measurements and FEM model simulation, we highlight the vertical displacement in Figure 13.

In Figure 13, the Y-coordinate is the centerline in the beam length direction, the Z-coordinate is the vertical displacement. The yellow line corresponds to the surface-based TLS measurement and blue line corresponds to the FEM model simulation. The TLS measurements (yellow line) show asymmetry on the two sides, most probably due to the torsion of the beam that could appear if, e.g., the support is not 100% horizontal. Another reason could be the measurement uncertainty of the TLS in compasion to the relative small deformations (0.35 mm). Additionally, the TLS is not fixed in height with respect to the beam. As mentioned above, the two ends of the beams are blocked by the hydraulic jack (see Figure 4), so the yellow line presents only the middle part of the beam which is in the scanning area of the TLS.

## 6. Conclusions

This paper compares TLS measurements with FEM model simulation of the load and displacement. The measurement accuracy and resolution of TLS are high enough for this application. Further research is necessary to reduce the errors in the data preprocessing, and the FEM model still needs more parameters to simulate more accurately and provide more reliable results. Nevertheless, it could be shown, that the surface-based measurements are very beneficial for the evaluation of FEM models.

We should remember that nowadays traffic is continuously increasing all of the world, especially in some developing countries [34,35]. According to statistics, in the past 10 years, the average growth rate of car sales, e.g., in China, has been between 30% and 40%. It will become more and more important to efficiently simulate the deformation of bridges and accurately predict their secure lifespan. For this reason, research in this field to provide a more powerful methodology and later also on practical software is very promising.

## Figures and Tables

**Table 1.** The measurement of load and displacement in epoch data [15].

**Epoch Data**	**1**	**2**	**3**	**4**	**5**	**6**	**7**	**8**	**9**
Load (kN)	0	14.17	22.40	31.85	37.10	43.60	54.52	58.92	65.85
Displacement (mm)	0	2.13	4.38	6.25	7.25	9.62	19.06	32.87	46.63

**Table 2.** Paramaters set of FEM model simulation.

**Materials**	**Material 1**						**Material 2**	
**Parameters**	**EX**	**PRXY**	**ShrCf-Op**	**ShrCf-Cl**	**UnTensSt**	**UnCompSt**	**EX**	**PRXY**
Value	3 × 10^4^ MPa	0.2	0.35	1	3.11 MPa	−1 MPa	2 × 10^5^ MPa	0.25
